# Interference-Aware Two-Level Differentiated Transmission for Improving Downlink Spatial Reuse in Dense WLANs

**DOI:** 10.3390/s22124429

**Published:** 2022-06-11

**Authors:** Lam Kwon, Eun-Chan Park

**Affiliations:** Department of Information and Communication Engineering, Dongguk University, Seoul 04620, Korea; lamk@dongguk.edu

**Keywords:** spatial reuse, carrier sensing, transmission power control, IEEE 802.11ax, WLAN

## Abstract

In this study, we address the problem of downlink throughput degradation in dense wireless local area networks (WLANs) based on the IEEE 802.11ax standard. We demonstrate that this problem essentially results from the asymmetric characteristic of carrier sense multiple access between downlink and uplink transmissions in infrastructure WLANs, and it is exacerbated by a dynamic sensitivity control algorithm that aims to improve spatial reuse (SR) in IEEE 802.11ax. To solve this problem, we propose the *interference-aware two-level differentiation* mechanism consisting of the *dual channel access* (DCA) and *supplemental power control* (SPC) schemes. The proposed mechanism introduces a new measure called a spatial reusability indicator, which roughly estimates the signal-to-interference ratio from the received signal strength of beacon frames. Based on this measure, stations (STAs) are classified into the following two categories: *spatial reusable STAs* (SR-STAs) and *non-spatial reusable STAs* (NSR-STAs). Because SR-STAs are more robust to interference than NSR-STAs, the DCA scheme prioritizes transmissions to SR-STAs over those to NSR-STAs by using differentiated carrier sensing thresholds. Moreover, the SPC scheme selectively increases the transmission power to NSR-STAs to compensate for transmission failure due to interference. By combining the SPC and DCA schemes, the proposed mechanism effectively differentiates the downlink transmissions to SR-STAs and NSR-STAs in terms of channel access and transmission power, and it can boost the possibility of successful SR. The proposed mechanism can be easily implemented in IEEE 802.11ax without any complex calculation or significant signaling overhead. Moreover, we provide a practical guideline to determine appropriate parameter values for use in the proposed mechanism. The extensive simulation results obtained in this study confirm that the proposed mechanism increases the downlink throughput by more than several times without decreasing the overall throughput, compared to the existing mechanisms, and it maintains fairness between SR-STAs and NSR-STAs in terms of the ratio of successful transmission.

## 1. Introduction

Wireless local area network (WLAN) is one of the most promising technologies that can provide high-rate and cost-effective wireless connectivity for mobile devices. The recent outburst of diverse smart devices and Internet of Things (IoT) applications has increased the density of the WLAN environment [1,2,3,4]. Since WLANs operate in a contention-based manner, significant interference is inevitable in dense WLANs. For this reason, it is challenging to provide satisfactory service to various devices and applications. The most up-to-date WLAN standard IEEE 802.11ax (called Wi-Fi 6) aims to improve the efficiency of WLANs in dense environments [5,6,7,8,9,10,11,12]. To this end, several new technologies have been introduced in IEEE 802.11ax. Its main features include multi-user multiple input multiple output for realizing simultaneous transmission to and from multiple devices, orthogonal frequency division multiple access for achieving efficient and flexible allocation of radio resources, target wake time for realizing high energy efficiency, and spatial reuse (SR) schemes for increasing network capacity through concurrent transmissions in multiple basic service sets (BSSs).

To improve the efficiency of dense WLANs, SR is the most important of the aforementioned techniques. The crucial point of SR is to increase the opportunity for simultaneous transmissions in multiple BSSs while suitably managing interference in overlapping BSSs (OBSSs). SR can be achieved by following several approaches, which can be classified into four categories [13]: tuning carrier sensing threshold (CST), transmission power control (TPC), rate adaptation, and directional antenna.

In this study, we focus on controlling carrier sensing and transmission power to enhance SR. In this approach, there occurs a fundamental trade-off between transmission opportunity and interference. As CST increases (i.e., the sensible area is narrowed), the number of transmission opportunities can increase at the cost of high interference. In order words, a high sensing threshold can alleviate the exposed node problem. However, if the CST is excessively high, the transmission is prone to fail due to interference. Conversely, a decreasing CST (i.e., widening the sensible area) is effective for avoiding interference, that is, the hidden node problem can be mitigated, but this limits the number of opportunities for simultaneous transmission. This trade-off cannot be completely avoided by adjusting the CST, and it is difficult to simultaneously solve the hidden node and exposed node problems [14,15]. A similar problem is encountered in the TPC approach. By controlling the transmission power, the probability of successful transmission can be increased, or interference can be mitigated. Increasing the transmission power helps increase the number of successful transmissions. However, a high transmission power not only increases the interference with on-going transmissions of neighboring nodes but also suppresses their transmission opportunities. Meanwhile, the transmission power and carrier sensing threshold affect each other. Their mutual interaction was investigated and it was demonstrated that the achievable network capacity depends on the ratio of transmission power to CST [16]. Consequently, it is impractical to determine or estimate the optimal values of CST or TPC because they change dynamically depending on various factors including network topology, number or density of nodes, and traffic load. Moreover, it is difficult to control the sensing threshold and transmission power in an integrated manner.

Another important issue must be considered for improving the SR performance in dense WLANs. In an infrastructure WLAN, a BSS consists of a single access point (AP) and multiple stations (STAs). Downlink (DL) transmission from the AP to the STAs is performed when the AP accesses the channel, while uplink (UL) transmission is realized when an individual STA accesses the channel. As long as all nodes (STAs and AP) have the same CST, they have comparable probabilities of channel access [10]. If the channel access mechanism is not suitably differentiated between the AP and STA, the chance of channel access by the AP for DL transmission decreases to be lower than that by all STAs for UL transmission [17,18]. As the WLAN becomes denser, that is, the number of STAs increases, the asymmetric channel access problem worsens.

The performance of SR can be further degraded when the dynamic sensitivity control (DSC) algorithm is applied without consideration of this asymmetric characteristic. According to the DSC algorithm, each STA dynamically adjusts its CST by measuring the received signal strength (RSS) of beacon frames transmitted periodically from an associated AP [19]. When STAs are located closer to their APs in multiple BSSs, they have higher CST (narrower sensing range), and therefore, they have more chances of concurrent transmission. On the contrary, an STA that is far from its AP has lower CST (wider sensing range), and its transmission is more suppressed to avoid interference from neighboring BSSs. By adjusting the CST value of each STA, the DSC algorithm can improve the UL throughput. Similarly, it can be applied to an AP for DL transmission [20]. The AP collects the RSS information of the data frames transmitted from all associated STAs. Its CST is adjusted based on the STA that has the lowest signal strength because it should detect all transmissions of the associated STAs. Unlike the DSC algorithm for UL transmission, the AP maintains a common value of CST, regardless of the destination STA. Therefore, if the DSC algorithm is applied to STAs and AP for UL and DL transmissions, the AP probably has a lower CST than the STAs, and it has a lower probability of channel access accordingly. This means that the asymmetric characteristic of DSC between DL and UL transmissions degrades the DL transmission performance.

Herein, we aim to improve the DL throughput in dense WLANs. We propose the *INterference-aware Two-level Differentiation* (INTD) mechanism consisting of the *dual channel access* (DCA) and *supplemental power control* (SPC) schemes. The core concept of INTD is as follows. First, we introduce a criterion for DL spatial reusability, which can be considered as the signal-to-interference ratio (SIR) measured by an STA in the OBSS area. Based on this criterion, we classify STAs into the following two categories: *spatial reusable STA* (SR-STA) and *non-spatial reusable STA* (NSR-STA). We consider that DL transmissions to SR-STAs are somewhat robust to inter-BSS interference whereas those to NSR-STA are susceptible. Accordingly, the DCA scheme differentiates the backoff procedure for transmissions to SR-STA and NSR-STA. This scheme allows aggressive transmissions to SR-STAs to achieve the desired increase in SR performance, whereas it makes the transmissions to NSR-STAs conservative to avoid transmission failure due to interference. By contrast, the SPC scheme is applied to increase the probability of successful transmissions to NSR-STAs. Even with the DCA scheme, transmissions to NSR-STAs cannot be protected adequately against interference because the STAs or APs of neighboring BSSs may not detect transmissions to NSR-STAs. To prevent this problem, the SPC scheme increases the transmission power when the AP transmits a data frame to an NSR-STA. This increase in power is effective in two ways: it increases the robustness to interference of transmissions to NSR-STAs and simultaneously suppresses the transmissions of interfering nodes. In this manner, the proposed INTD mechanism effectively combines the approaches of carrier sensing and TPC, and it can significantly improve the DL throughput in dense WLANs while mitigating severe interference. The simulation results obtained in this study confirm that the INTD mechanism increases the DL throughput by more than several times compared to those of the existing mechanisms, and it maintains fairness between SR-STAs and NSR-STAs in terms of the ratio of successful transmission. The contributions of this paper are summarized as follows:

- We investigate the fundamental trade-off between transmission opportunity and interference that arises when controlling carrier sensing threshold and transmission power to enhance SR in dense WLANs. Moreover, we demonstrate that the DL throughput is considerably degraded because of the asymmetric characteristics between DL and UL transmissions.
- By introducing a DL spatial reusability indicator, we propose the integrated two-level differentiation mechanism between SR-STAs and NSR-STAs in terms of carrier sensing and transmission power. The proposed mechanism effectively improves the DL throughput without decreasing the overall throughput and without deteriorating fairness between SR-STAs and NSR-STAs.
- The proposed mechanism can be implemented easily without complex calculations and significant signaling overheads. In addition, we provide a practical design guideline for determining the parameters of the proposed mechanism.

The remainder of this paper is organized as follows. In Section 2, we state the novelty and contribution of our study by discussing and comparing it with related studies. Next, we propose the INTD mechanism and address how to appropriately determine its parameters in Section 3. We evaluate and compare the performance of the proposed mechanism by conducting an extensive simulation study in Section 4. Our conclusions are presented in Section 5.

## 2. Related Work

SR is a well-known issue in WLANs based on carrier-sense multiple access (CSMA), and many studies have been conducted to improve SR. We focus on two conventional approaches for improving SR in terms of carrier sensing and power control. Moreover, we investigate the SR schemes recently introduced in the IEEE 802.11ax standard.

First, several studies [21,22,23,24,25,26,27] have aimed to improve SR by controlling CST or determining its optimal value. In [21], an analytical model was presented to determine the optimal value of CST that can maximize the number of successful transmissions in multi-hop mesh networks. This theoretical study was conducted under the assumption that the interference model and network topology are given. To avoid complex computations and requirements for obtaining the optimal CST, in [22], a heuristic algorithm was proposed, in which the parameters required for CST tuning can be estimated practically. In [23], the authors analyzed the effects of CST and backoff mechanism on network throughput and collision probability in 802.11 ad hoc networks and developed an analytical model for CST optimization. In [24], the causes of transmission failure were classified into collision and interference, and a method was proposed to statistically estimate the probability of collision and interference. Based on this differentiation, the authors proposed a centralized algorithm for CST adaptation. Furthermore, to mitigate the hidden/exposed node problems, the study in [25] proposed a method for each STA to adaptively select its CST based on busy or idle signals broadcast periodically by an AP. In [26], a combined algorithm for CST control and AP selection was proposed. This algorithm considered co-channel interference and traffic load to achieve the maximum throughput in dense WLANs. In [27], the authors proposed to include the CST value required to protect its transmission in the preamble of a frame, such that the neighboring nodes can transmit only when their own and ongoing transmissions will succeed, and they proposed model-based and measurement-based schemes to calculate the CST value.

Second, in [28,29,30,31,32], TPC is employed as a means for improving SR. These studies attempted to increase the number of successful parallel transmissions by suppressing interference from neighboring nodes. In [28], the authors analyzed the relationship among the transmission range, carrier detection range, and interference range when the TPC scheme was adopted, and they proposed four adaptive range-based power control mechanisms to avoid collisions in wireless ad hoc networks. Similar to the ready-to-send/clear-to-send exchange, the request-power-to-send/acceptable-power-to-send handshake mechanism was proposed in the power controlled multiple access protocol [29] to determine the minimum transmission power for successful packet reception at the receiver. In [30], it was demonstrated that power control may worsen the hidden terminal problem, and the collision avoidance power control mechanism was proposed to solve this problem by determining an appropriate transmit power considering the interference range. In [31], STAs were divided into BSS-edge STAs and BSS-center STAs, and fractional CSMA was proposed to adjust the transmission power or channel access depending on the group of STAs. To reduce channel interference, a dynamic TPC algorithm was proposed in [32]; it works based on real-time measurements of channel occupancy and wireless link status without exchanging signaling information between the AP and the associated STAs or between neighboring APs.

Recently, in IEEE 802.11ax, two schemes were standardized to improve SR; OBSS packet detection (OBSS PD) and parameterized spatial reuse (PSR) [5,9]. The OBSS PD-based SR operation distinguishes an inter-BSS transmission from an intra-BSS transmission. For this purpose, the BSS color information contained in the physical layer header is used to identify the BSS of a data frame [33]. Meanwhile, intra-BSS transmissions are detected using a conservative CST (lower CST) to avoid interference within the BSS, and inter-BSS transmissions are detected using an aggressive CST (higher CST) to allow concurrent transmission among multiple BSSs. The STA determines the CST value for inter-BSS detection, referred to as OBSS PD level, by considering the transmission power, received power of beacon frame, and channel bandwidth. Moreover, the transmission power of inter-BSS transmission can be limited depending on the OBSS PD level [5]. On the other hand, the PSR-based SR operation uses a trigger-based transmission, which is newly adopted in IEEE 802.11ax, for scheduled channel access. The AP determines the availability of SR, along with the transmission duration and power, and provides this information to the STA through a trigger frame. Upon receiving the trigger frame, the STA can initiate the SR operation for the specified duration. Moreover, it is possible for the AP to execute the SR operation after detecting the trigger frames of neighboring BSSs. The IEEE 802.11ax standard establishes the framework and signaling for SR operations; however, the detailed mechanism or procedure is out of the scope of this standard and depends on the specific implementation.

The conventional SR mechanisms addressed above mainly consider ad hoc networks or mesh networks, whose characteristics are rather different from those of overlappingly deployed infrastructure WLANs. The mechanisms described in the literature are somewhat applicable for improving UL SR, but they cannot be used to solve the problem of DL performance degradation due to the asymmetry between DL and UL transmissions. As a network becomes denser with multiple nodes, interference is exacerbated, and signaling between the AP and STAs to estimate or handle interference incurs enormous overheads. Therefore, the existing approach of dynamically adjusting the CST or transmission power is neither practical nor desirable in dense WLANs.

## 3. Interference-Aware Two-Level Differentiation Mechanism

In this section, we introduces the INTD mechanism consisting of the DCA and SPC schemes. First, we discuss how to divide the STAs into SR-STAs and NSR-STAs by considering interference in the OBSS area. Then, we describe two differentiation schemes, namely DCA and SPC. Finally, we explain how to properly set the parameters values in the INTD mechanism.

### 3.1. Station Classification Based on Spatial Reusability

When an AP transmits a frame to a certain STA, the probability that the STA can correctly receive the frame depends primarily on the signal-to-interference-and-noise ratio (SINR). When the SINR is high, the probability of successful delivery of a frame is higher. Thus, concurrent transmission by the AP may be allowed so long as the STA receives the frame with a sufficiently high SINR, even if there is an ongoing transmission near the receiving STA. That is, SR depends mainly on SINR. However, it is difficult for the AP to estimate the SINR value in an accurate and timely manner in a CSMA-based WLAN without explicit feedback from the STA to the AP. To solve this problem, we introduce a new criterion for spatial reusability. It can be measured using the RSS of the beacon frames (BFs) transmitted by several APs without requiring an additional signaling mechanism.

According to the IEEE 802.11 standard, an AP periodically broadcasts BFs to advertise its BSS identifier and manage the network. In dense WLANs, an STA may receive BFs from several APs belonging to different BSSs. Let us define $P_{BF(i,j)}$ as the RSS (in dBm) of the BF transmitted by ${\mathrm{AP}}_{i}$ and measured by ${\mathrm{STA}}_{j}$. Moreover, we define $S_{j}$ as the set of APs whose BFs can be correctly received by ${\mathrm{STA}}_{j}$, $k(\in S_{j})$ as the index of AP with which ${\mathrm{STA}}_{j}$ attempts to associate, and $k^{\prime }\left(\in S_{j}\right)$ as that of AP whose BF has the highest RSS in ${\mathrm{STA}}_{j}$ except for *k*. We propose that during the association procedure, ${\mathrm{STA}}_{j}$ measures $P_{BF(k,j)}$ and $P_{BF(k^{\prime },j)}$ and sends an association request message to ${\mathrm{AP}}_{k}$ along with them; then, ${\mathrm{AP}}_{k}$ calculates the *spatial reusability indicator* of ${\mathrm{STA}}_{j}$ (denoted as $SRI_{\left(j\right)}$) as follows:

(1) $$SRI_{\left(j\right)}=P_{BF(k,j)}-P_{BF(k^{\prime },j)}.$$

As shown in (1), $SRI_{\left(j\right)}$ can be considered the approximated SIR measured by the ${\mathrm{STA}}_{j}$ located in the OBSS area when two adjacent APs transmit frames concurrently. However, we would like to emphasize that SRI is introduced not to measure SIR accurately but to determine the spatial reusability practically. Instead of reporting $P_{BF(k,j)}$ and $P_{BF(k^{\prime },j)}$ to ${\mathrm{AP}}_{k}$, ${\mathrm{STA}}_{j}$ can calculate $SRI_{\left(j\right)}$ from (1) and inform ${\mathrm{AP}}_{k}$ of its SRI value. Moreover, the process of the SRI report can be extended to cope with station mobility. The STA updates the SRI value when it receives the BFs that APs broadcast periodically. If there is a great change in the SRI value, the STA reports it to the associated AP using a control frame or piggybacking on a data frame. It is possible that ${\mathrm{STA}}_{j}$ receives the BF only from ${\mathrm{AP}}_{k}$, that is, there are no interfering neighbor APs. In this case, $P_{BF(k^{\prime },j)}$ can be replaced with the CST of the legacy device (e.g., −82 dBm with the channel bandwidth of 20 MHz). We define $SRI_{TH}$ as a threshold for determine the spatial reusability. By comparing $SRI_{j}$ and $SRI_{TH}$, ${\mathrm{STA}}_{j}$ is classified as an SR-STA if $SRI_{j}>SRI_{TH}$ or as an NSR-STA otherwise.

### 3.2. Dual Channel Access Scheme

We designed the DCA scheme to improve SR by providing more DL transmission opportunities to SR-STAs than to NSR-STAs. A simultaneous DL transmission to an SR-STA may succeed with a higher probability than that to an NSR-STA because the former is less susceptible to the interference than the latter. To prioritize transmissions to SR-STAs over those to NSR-STAs, the DCA scheme uses two differentiated values of CST for SR-STAs and NSR-STAs, and they are denoted as $CST_{SR}$ and $CST_{NSR}$, respectively. Moreover, we denote SR and NSR frames as those destined to SR-STAs and NSR-STAs, respectively.

**Figure 1:**
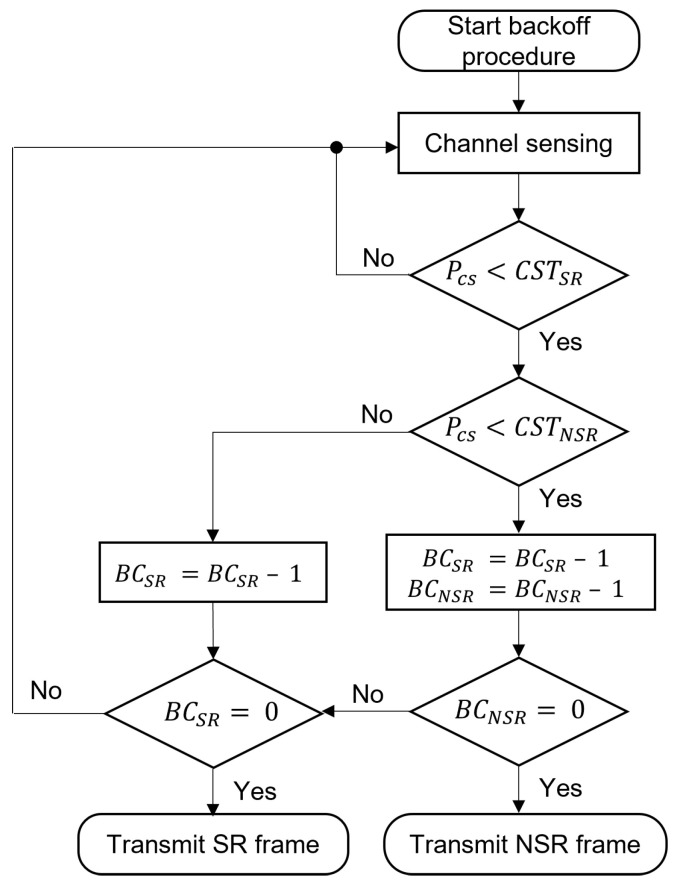
Flowchart of backoff procedure in dual channel access scheme.

The flowchart in Figure 1 shows the backoff operation of DCA with dual CST. Note that $CST_{SR}$ is set to be greater than $CST_{NSR}$, and the AP maintains two backoff counters $BC_{SR}$ and $BC_{NSR}$ for the SR and NSR frames, respectively, as well as two separate queues. As shown in Figure 1, if the AP determines that the carrier sensing power ($P_{CS}$) is lower than $CST_{NSR}$, both backoff counters are decreased by one. Otherwise, if $P_{CS}$ is greater than $CST_{NSR}$, but lower than $CST_{SR}$, $BC_{SR}$ is decreased. When the backoff procedure is completed, that is, when $BC_{SR}$ or $BC_{NSR}$ becomes zero, the AP transmits SR or NSR frames, respectively. If both $BC_{SR}$ and $BC_{NSR}$ become zero at the same time, the NSR frame is transmitted because it has fewer opportunities than the SR frame. The DCA scheme manages two binary exponential backoff mechanisms for SR and NSR frames, that is, the contention window for NSR frames is doubled while that for SR frames is not changed if transmission of the NSR frame fails.

**Figure 2:**
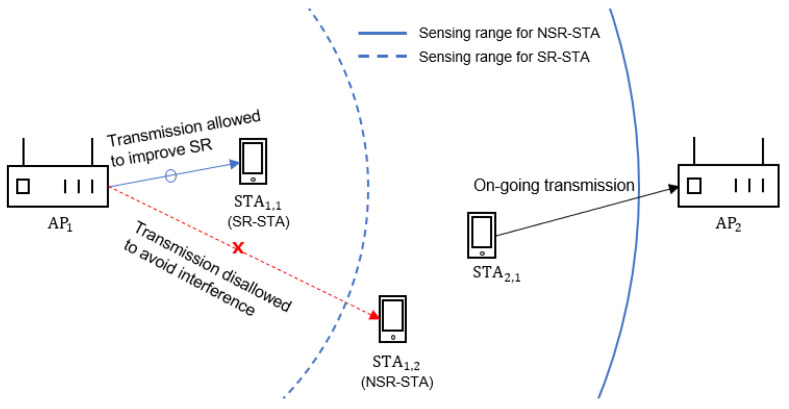
Operational example of spatial reuse by the dual channel access scheme.

Figure 2 illustrates an operational example of the DCA scheme. Here, ${\mathrm{STA}}_{i,j}$ denotes the *j*-th STA associated with ${\mathrm{AP}}_{i}$. In Figure 2, ${\mathrm{STA}}_{1,1}$ and ${\mathrm{STA}}_{1,2}$ are associated with ${\mathrm{AP}}_{1}$ and classified as SR-STA and NSR-STA, respectively. Assume that ${\mathrm{STA}}_{2,1}$ is transmitting a data frame to ${\mathrm{AP}}_{2}$. During the transmission time of ${\mathrm{STA}}_{2,1}$, ${\mathrm{AP}}_{1}$ can continue the backoff procedure for ${\mathrm{STA}}_{1,1}$, but not for ${\mathrm{STA}}_{1,2}$ because ${\mathrm{AP}}_{1}$ does not detect the transmission of ${\mathrm{STA}}_{2,1}$ with $CST_{SR}$, but detects it with $CST_{NSR}$. Consequently, ${\mathrm{AP}}_{1}$ can transmit to SR-STA (${\mathrm{STA}}_{1,1}$) and the exposed node problem can be alleviated. Notably, in addition to the enhancement of SR, the DCA scheme is effective for reducing transmission failure due to interference. In this example, the DCA scheme prevents ${\mathrm{AP}}_{1}$ from transmitting to NSR-STA (${\mathrm{STA}}_{1,2}$) for the transmission duration of ${\mathrm{STA}}_{\left(2,1\right)}$.

### 3.3. Supplemental Power Control for NSR-STA

The SPC scheme aims to increase the probability of successful transmission to NSR-STAs without impairing the possibility of SR. Transmission to NSR-STAs is more susceptible to interference and is more likely to fail. Its failure not only causes a waste of channel resources, but also worsens fairness between SR-STAs and NSR-STAs. Because the binary exponential backoff mechanisms for SR-STAs and NSR-STAs work independently, frequent transmission failures to NSR-STAs increase the contention window for NSR-STAs, but they do not affect that for SR-STAs. Thus, the service opportunities available to NSR-STAs further decrease. The SPC scheme solves this problem by increasing the transmission power selectively when the AP transmits to NSR-STAs.

**Figure 3:**
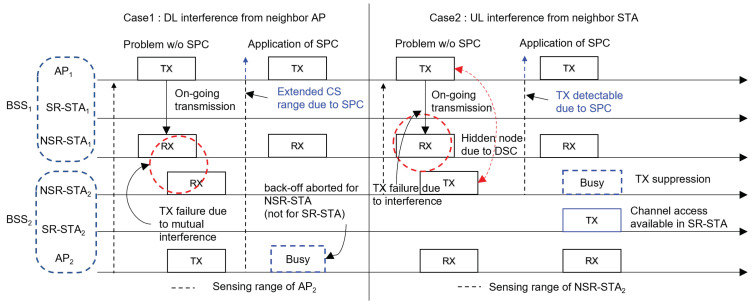
Two operational examples of supplemental power control scheme.

Figure 3 shows how the SPC scheme can decrease the number of transmission failures of NSR frames and suppress the transmissions of interfering neighboring nodes. Here, we consider that two BSSs are partially overlapped, and each BSS consists of an AP, one SR-STA, and one NSR-STA. Moreover, we consider two cases: Case 1, in which transmission of the NSR frame is subjected to interference from a neighboring AP, and Case 2, in which interference occurs because of a neighboring NSR-STA.

In Case 1, ${\mathrm{AP}}_{1}$ transmits a frame to ${\mathrm{NSR}\text{-}\mathrm{STA}}_{1}$ and ${\mathrm{AP}}_{2}$ is unaware of this transmission (i.e., hidden from ${\mathrm{AP}}_{1}$) and attempts to transmit to ${\mathrm{NSR}\text{-}\mathrm{STA}}_{2}$. In this case, both transmissions probably fail because of high mutual interference. The SPC scheme can avoid this scenario by increasing the transmission power of ${\mathrm{AP}}_{1}$ such that ${\mathrm{AP}}_{2}$ can detect the transmission of ${\mathrm{AP}}_{1}$ and cease its backoff procedure until the end of ${\mathrm{AP}}_{1}$’s transmission. It is worthwhile to note that the SPC scheme only blocks the simultaneous transmission of NSR frames by ${\mathrm{AP}}_{1}$ and ${\mathrm{AP}}_{2}$. For example, when ${\mathrm{AP}}_{1}$ transmits to ${\mathrm{SR}\text{-}\mathrm{STA}}_{1}$, the SPC scheme is not applied, meaning that the transmission of ${\mathrm{AP}}_{2}$ is not blocked, regardless of whether its destination is ${\mathrm{SR}\text{-}\mathrm{STA}}_{2}$ or ${\mathrm{NSR}\text{-}\mathrm{STA}}_{2}$. Furthermore, even when ${\mathrm{AP}}_{1}$ is transmitting to ${\mathrm{NSR}\text{-}\mathrm{STA}}_{1}$, the combination of the SPC and DCA schemes does not prevent ${\mathrm{AP}}_{2}$ from transmitting to ${\mathrm{SR}\text{-}\mathrm{STA}}_{2}$ because of the higher CST of SR-STAs.

Next, we consider Case 2 in which ${\mathrm{NSR}\text{-}\mathrm{STA}}_{2}$ cannot detect the transmission of ${\mathrm{AP}}_{1}$ to ${\mathrm{NSR}\text{-}\mathrm{STA}}_{1}$. This case may occur when the inter-BSS transmission is ignored by the OBSS PD mechanism in the 802.11ax standard or when the CST of STA increases because of the DSC algorithm. In this case, the transmission of ${\mathrm{NSR}\text{-}\mathrm{STA}}_{2}$ interferes severely with that to ${\mathrm{NSR}\text{-}\mathrm{STA}}_{1}$, which is located close to ${\mathrm{NSR}\text{-}\mathrm{STA}}_{2}$, and increases the probability that the transmission of ${\mathrm{AP}}_{1}$ to ${\mathrm{NSR}\text{-}\mathrm{STA}}_{1}$ fails. However, if the SPC scheme is applied, ${\mathrm{NSR}\text{-}\mathrm{STA}}_{2}$ can detect the transmission of ${\mathrm{AP}}_{1}$ so its own transmission is suppressed, but channel access by ${\mathrm{SR}\text{-}\mathrm{STA}}_{2}$ may not be blocked due to the DSC algorithm.

### 3.4. Design Guideline for Parameters of INTD Mechanism

The proposed INTD mechanism has three key parameters: (i) $SRI_{TH}$, the threshold of the SRI for classifying STAs, (ii) $CST_{SR}$, the carrier sensing threshold of SR-STAs in the DCA scheme, and (iii) $\Delta P_{tx}$, the additional transmission power of NSR-STAs in the SPC scheme. In this subsection, we describe how to properly set these parameters. For tractability, we make the following assumptions. The BSSs are deployed in a honeycomb structure with a central BSS and six BSSs surrounding the central BSS, and the distance between two adjacent APs is fixed. APs have the same transmission power and an STA attempts to associate with the AP whose BF has the strongest RSS. The RSS is determined using a log-distance path-loss model without considering the effect of fading. The modulation and coding scheme (MCS) is fixed, that is, rate adaptation is not considered.

#### 3.4.1. Threshold of Spatial Reusability Indicator

**Figure 4:**
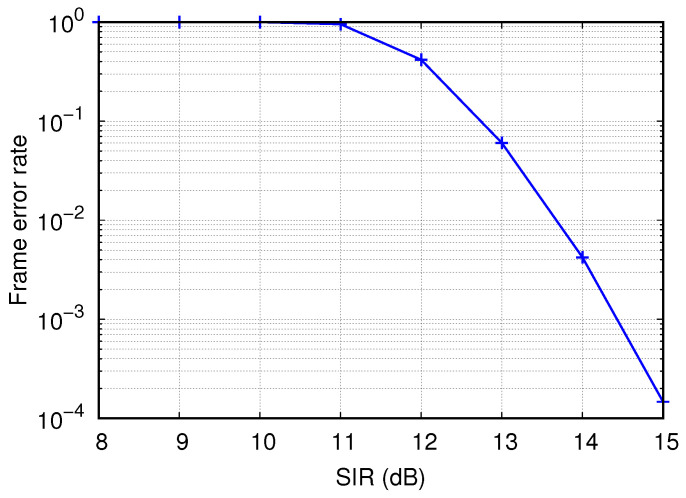
Frame error rate depending on signal-to-interference ratio.

We set $SRI_{TH}$ such that the transmission of an SR frame is probably successful, even with a concurrent DL transmission from a neighboring BSS. Because SRI in (1) is roughly similar to the SIR value in this case, $SRI_{TH}$ is determined to ensure that the frame error rate (FER) associated with transmission of the SR frame is lower than an acceptable level. By using the simulation scenario and performance evaluation methodology of IEEE 802.11ax [34,35], we can obtain the symbol error rate (SER) for a given MCS, frame size, and SINR [36]. Then, the FER can be calculated as follows:

(2) $$FER=1-{\left(1-SER\right)}^{N_{S}},$$

where $N_{S}$ is the number of symbols in the frame. In calculating the FER value from (2), we use SIR instead of SINR because the noise power is insignificant compared to interference power in dense networks. Figure 4 shows the FER value obtained from (2) versus the SIR value when the MCS is quadrature phase shift keying (QPSK) 3/4 and the frame size is 1472 bytes in outdoor scenarios of IEEE 802.11ax [34]. As shown in Figure 4 the FER is almost close to one so long as the SIR is smaller than a certain value (e.g., 11 dB), but it decreases rapidly as the SIR increases. In this study, we set $SRI_{TH}$ as 13 dB to ensure that the FER is lower then 10%.

#### 3.4.2. CST for SR-STAs in DCA Scheme

The higher $CST_{SR}$ is, the stronger is the preference of the AP to transmit SR frames than NSR frames. However, an excessively high $CST_{SR}$ value causes transmission failures due to severe interference. Based on this point, we can determine the upper bound of $CST_{SR}$. We consider the honeycomb structure of BSSs and focus on the central BSS. We define $D_{AP}$ as the distance between any two adjacent APs located at the center of each BSS. Under the assumption that an STA associates with the AP having the highest signal strength, the minimum distance between the AP in the central BSS and any STA in the neighboring BSSs is half of $D_{AP}$. We consider the following log-distance path-loss model developed for the TGax outdoor large BSS scenario [34]:

(3) PL(d)=36.7log(d)+26.0log(f)+22.7(dB),

where *d* (m) is the distance between source and destination nodes, and *f* (GHz) is the frequency of the wireless channel. By using this path-loss model and the minimum distance between the central AP and the inter-BSS STA, we can determine the maximum interference power ($P_{I}^{max}$ in dBm) that may affect the transmission by the central AP as follows:

(4) $$P_{I}^{max}=P_{TX}-PL\left(\frac{D_{AP}}{2}\right),$$

where $P_{TX}$ is the transmission power of the interfering STA. If $CST_{SR}$ is higher than $P_{I}^{max}$, the central AP can transmit to the SR-STA while the interfering inter-BSS STA is transmitting. The transmission of AP can be successful so long as the SR-STA is located far from the inter-BSS STA. However, the AP can only measure the carrier sensing power, but is unaware of the locations of SR-STA or interfering STA. When the maximum interference occurs by all neighboring STAs in the six BSSs surrounding the central BSS, it is desirable to not access the channel because transmissions from the central AP to any SR-STA are rarely successful. By considering this worst-case interference, we can set the upper bound of $CST_{SR}$ ($CST_{SR}^{max}$ in dBm) as follows:

(5) $$CST_{SR}^{max}=10\log\left(6\right)+P_{I}^{max},$$

that is, transmission of the SR frame is permitted if $P_{CS}$ does not exceed $CST_{SR}^{max}$. For example, when $P_{TX}$ is 25 dBm and $D_{AP}$ is 80 m, $CST_{SR}^{max}$ is −66 dBm. Note that as specified in the IEEE 802.11 standard, we set $CST_{NSR}$ to −82 dBm to maintain backward compatibility with legacy WLANs.

#### 3.4.3. Transmission Power of NSR-STA in SPC Scheme

When setting the transmission power in the SPC scheme, it is essential to achieve a balance between successful transmission to NSR-STAs and the possibility of SR with SR-STAs. We define $P_{TX}^{+}=P_{TX}+\Delta P_{SPC}$ (dBm) as the transmission power to NSR-STAs. Note that $\Delta P_{SPC}\left(>0\right)$ (dBm) is added only for NSR frame transmission, and $P_{TX}$ is used for SR frame transmission. Moreover, we define $P_{RX}\left(d\right)$ (dBm) as the RSS when the distance between the sender and receiver is *d* (m), which can be represented using the path-loss model in (3) as $P_{RX}\left(d\right)=P_{TX}^{+}-PL\left(d\right)$ in the case of NSR frame transmission. By considering Case 1 of Figure 3, we can determine the condition for $P_{RX}$ as follows.

(6) $$CST_{NSR}<P_{RX}\left(D_{AP}\right)=P_{TX}^{+}-PL\left(D_{AP}\right)<CST_{SR}.$$

We can rewrite (6) in terms of $\Delta P_{SPC}$ as

(7) $$CST_{NSR}-P_{TX}+PL\left(D_{AP}\right)<\Delta P_{SPC}<CST_{SR}-P_{TX}+PL\left(D_{AP}\right).$$

The condition of $\Delta P_{SPC}$ in Case 2 can be obtained in a similar way to that in Case 1. The transmission of ${\mathrm{AP}}_{1}$ to ${\mathrm{NSR}\text{-}\mathrm{STA}}_{1}$ should be detected by ${\mathrm{NSR}\text{-}\mathrm{STA}}_{2}$ to avoid interference, but channel access by ${\mathrm{SR}\text{-}\mathrm{STA}}_{2}$ needs to be allowed to improve SR. In contrast to Case 1, we need to consider the DSC algorithm, which adjusts the CST value for UL transmission of STAs. We define $CST_{UL}\left(d\right)$ as the CST value determined by the DSC algorithm, where *d* is the distance between the STA and its associated AP. According to the DSC algorithm, $CST_{UL}\left(d\right)$ is mainly determined based on the average RSS of the BFs that are transmitted from the associated AP. Similar to (7), the lower bound of $\Delta P_{SPC}$ in Case 2 can be obtained when ${\mathrm{NSR}\text{-}\mathrm{STA}}_{2}$ is the farthest from ${\mathrm{AP}}_{2}$ ($d=D_{AP}/2$), that is,

(8) $$\Delta P_{SPC}>CST_{UL}\left(\frac{D_{AP}}{2}\right)-P_{TX}+PL\left(\frac{D_{AP}}{2}\right).$$

Now, we focus on the upper bound of $\Delta P_{SPC}$. We define $d_{SR}^{max}$ as the maximum distance between ${\mathrm{SR}\text{-}\mathrm{STA}}_{2}$ and ${\mathrm{AP}}_{2}$, which can be obtained using (1) and $SRI_{TH}$. Then, the upper bound of $\Delta P_{SPC}$ can be determined as

(9) $$\Delta P_{SPC}<CST_{UL}\left(d_{SR}^{max}\right)-P_{TX}+PL\left(D_{AP}-d_{SR}^{max}\right).$$

Note that the first term $CST_{UL}$ on the right side of (9) considers the distance between ${\mathrm{SR}\text{-}\mathrm{STA}}_{2}$ and ${\mathrm{AP}}_{2}$ while the third term PL considers the distance between ${\mathrm{SR}\text{-}\mathrm{STA}}_{2}$ and ${\mathrm{AP}}_{1}$.

The condition of $\Delta P_{SPC}$ can be obtained numerically from (7)–(9). For example, when $CST_{NSR}$ = −82 dBm, $CST_{SR}$ = −67 dBm, $P_{TX}$ = 25 dBm, $SRI_{TH}$ = 13 dB, $D_{AP}$ = 80 m, and $d_{SR}^{max}$ = 24 m, the condition of $\Delta P_{SPC}$ in Case 1 is 3 dBm $<\Delta P_{SPC}<$ 18 dBm and that in Case 2 is 0 dBm $<\Delta P_{SPC}<$ 15 dBm. Based on these two cases, the value of $\Delta P_{SPC}$ lies between 3 dBm and 15 dBm. The effect of $\Delta P_{SPC}$ will be evaluated via simulation in the next section.

## 4. Simulation Results

In this section, we evaluate the performance of the proposed INTD mechanism by conducting extensive simulations. We implemented a simulator using MATLAB by considering the operations of IEEE 802.11ax and complying with the simulation methodology and scenario described in [34,35]. We performed the simulation with the honeycomb topology consisting of a central BSS and six BSSs surrounding the central BSS, except for random deployment of BSSs, and focused on the performance of the central BSS. In each simulation instance, STAs were placed at random locations, and each STA was associated with the closest AP. The simulation was repeated 50 times to obtain the average value or distribution. We assumed that all nodes, including APs, always have data frames to transmit, and the frame size and MCS were fixed. Table 1 lists several of the simulation parameters. We considered the following mechanisms for performance comparison:

- BASE: This is the baseline mechanism without the adoption of any technique to improve SR.
- DSC-UL: In this mechanism, the DSC algorithm is implemented only in the STAs for UL transmission, as proposed in [19]. The margin was set as zero to maximize the effect of UL SR.
- DSC-DL: The DSC algorithm is implemented in both APs and STAs for DL and UL transmissions [20].
- INTD(DCA): The proposed DCA scheme is implemented in the APs for differentiated DL transmissions to SR-STAs and NSR-STAs. The DSC algorithm is also implemented in the same way as that in the DSC-UL mechanism.
- INTD(DCA&SPC): This is the proposed mechanism consisting of the DCA and SPC schemes. Compared to INTD(DCA), the SPC scheme is additionally implemented to focus on its effect.

We performed extensive simulations as follows. First, we focus on DL throughput degradation due to the asymmetric behavior of dense WLANs in Section 4.1. Next, we evaluate the effect of SPC and determine the optimal value of $\Delta P_{SPC}$ through simulations in Section 4.2. In Section 4.3 and Section 4.4, we compare the performance of several mechanisms in terms of various aspects in depth. Finally, we investigate the fairness issue between SR-STAs and NSR-STAs in Section 4.5.

### 4.1. Downlink Throughput Degradation

**Figure 5:**
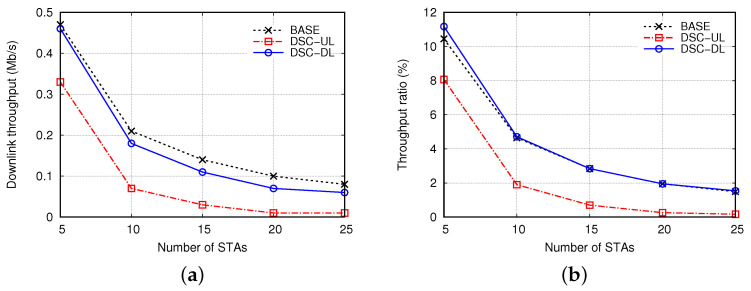
Comparison of (**a**) downlink throughput and (**b**) its ratio with respect to number of stations.

We define $TH_{D}$ and $TH_{U}$ as the DL and UL throughputs achieved by the AP and all STAs in the central BSS, respectively, and $TH_{T}$ as the total throughput, that is, $TH_{T}=TH_{D}+TH_{U}$. Moreover, we define $R_{D}^{TH}$ as the ratio of DL throughput to total throughput, that is, $R_{D}^{TH}=TH_{D}/TH_{T}$. Figure 5 shows $TH_{D}$ and $R_{D}^{TH}$ when the distance between APs ($D_{AP}$) was 80 m and the number of STAs per BSS ($N_{STA}$) was increased from 5 to 25. Owing to severe interference in the dense OBSS environment, the asymmetric properties of channel access and DSC algorithm between the DL and UL transmissions, $TH_{D}$ decreased sharply as $N_{STA}$ increased, and $R_{D}^{TH}$ did not exceed 5% in most cases. The problem of DL throughput degradation deteriorated in DSC-UL. Compared to BASE, $TH_{D}$ decreased several times in DSC-UL, and $R_{D}^{TH}$ was lower than 2%, except in the case when $N_{STA}$ = 5. This was because the AP was further deprived of DL transmission opportunities, especially when the DSC algorithm was applied only to the STAs for UL transmission. This problem was somewhat alleviated in DSC-DL, and $TH_{D}$ and $R_{D}^{TH}$ were comparable to those in BASE. However, $R_{D}^{TH}$ decreased from 11% to 1.5% when $N_{STA}$ increased from 5 to 25. These results confirm that the DL throughput is degraded significantly in dense WLANs, and it is critical to solve this problem.

### 4.2. Effect of Supplemental Power Control

**Figure 6:**
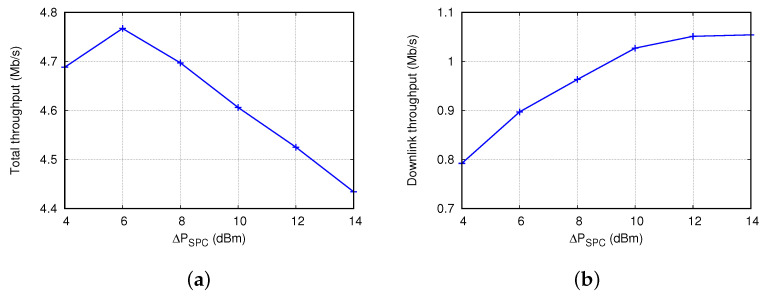
Effect of additional transmission power in the supplemental power control scheme ($\Delta P_{SPC}$) on (**a**) total throughput and (**b**) downlink throughput.

Figure 6 shows how the supplemental transmission power ($\Delta P_{SPC}$) affects $TH_{T}$ and $TH_{D}$ in INTD(DCA&SPC). Here, $N_{STA}$ and $D_{AP}$ were set to 10 and 80 m, respectively. We increased $\Delta P_{SPC}$ from 4 dBm to 14 dBm based on the analysis results of its condition in Section 3.4.3. As shown in Figure 6a, $TH_{T}$ was maximized when $\Delta P_{SPC}$ = 6 dBm, and it decreased almost linearly when $\Delta P_{SPC}$ > 6 dBm. A large $\Delta P_{SPC}$ increases the probability of successful transmission to NSR-STAs, but it blocks SR opportunities. The result in Figure 6a validates this trade-off. By contrast, $TH_{D}$ increased as $\Delta P_{SPC}$ increased from 4 dBm to 12 dBm, and it was almost constant when $\Delta P_{SPC}>$ 12 dBm. Larger values of $\Delta P_{SPC}$ were helpful for improving DL throughput because more inter-BSS STAs were blocked to avoid interference, and the AP had more chances for successful simultaneous transmission. The best values of $\Delta P_{SPC}$ were approximately 6 dBm and 12 dBm for $TH_{T}$ and $TH_{D}$, respectively. When $\Delta P_{SPC}$ increased from 6 dBm to 10 dBm, $TH_{D}$ increased by 15% but $TH_{T}$ decreased by only 3.5%. Hereinafter, we set $\Delta P_{SPC}$ to 10 dBm to strike a balance between the increase in $TH_{D}$ and decrease in $TH_{T}$.

### 4.3. Performance Comparison of Several Mechanisms

**Figure 7:**
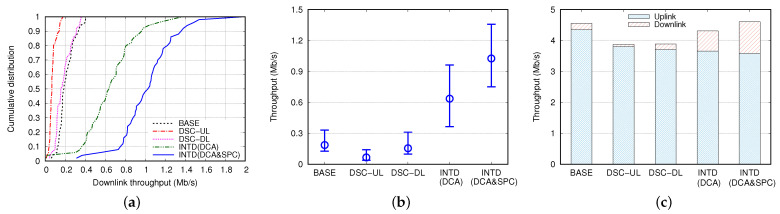
Performance comparison of several mechanisms in terms of (**a**) distribution of downlink throughput; (**b**) median, 10th, and 90th percentiles of downlink throughput; and (**c**) uplink and downlink throughputs.

Now, we compare the performance of the proposed mechanism with several mechanisms in terms of various aspects. Figure 7a shows a cumulative distribution of $TH_{D}$ obtained from 50 simulations when $N_{STA}$ = 10 and $D_{AP}$ = 80 m. In general, INTD(DCA&SPC) achieved the highest $TH_{D}$, which was greater than that achieved by INTD(DCA) by 40–80%; difference between the $TH_{D}$ values of DSC-DL and BASE was insignificant; and DSC-UL performed the worst in terms of $TH_{D}$. The middle circle on the line in Figure 7b indicates the median value of $TH_{D}$, and both ends of line represent the 10th and 90th percentile values of $TH_{D}$. Compared to the values obtained in the BASE case, the median values of $TH_{D}$ in DSC-UL and DSC-DL decreased by approximately 65% and 13%, respectively, but increased by 3.4 and 5.5 times in the cases of INTD(DCA) and INTD(DCA&SPC), respectively. Compared to INTD(DCA), the 10th percentile, median, and 90th percentile of $TH_{D}$ increased in the case of INTD(DCA&SPC) by 106%, 62%, and 41%, respectively. Moreover, $TH_{U}$, $TH_{D}$, and $TH_{T}$ values can be observed from Figure 7c. These values were obtained by averaging the results of 50 simulations. There were few differences in $TH_{U}$ and $TH_{T}$ between DSC-UL and DSC-DL, and these $TH_{U}$ and $TH_{T}$ values were smaller those obtained in the BASE case by approximately 10% and 12%, respectively. In the cases of INTD(DCA) and INTD(DCA&SPC), $TH_{U}$s decreased by 16% and 18%, respectively, compared to that in the BASE case. However, in the case of INTD(DCA&SPC), the $TH_{T}$ value was marginally higher than that in BASE, while the $TH_{D}$ value was approximately 5 times higher than that in BASE (see Figure 7a,b). We can summarize the main results depicted in Figure 7 as follows.

- Compared to the BASE case, DSC-UL worsened the performance in terms of both DL and UL throughputs. This stems mainly from the excessive channel access by STAs and transmission failure due to severe interference.
- Application of the DSC algorithm to DL transmission in DSC-DL was somewhat helpful for increasing the DL throughput compared to that in DSC-UL. However, the effectiveness of DSC-DL was marginal; it achieved a DL throughput comparable to that in the BASE case, but its UL throughput was rather smaller than that in the BASE case.
- Owing to the differentiated transmission based on the DCA scheme, INTD(DCA) greatly improved the DL throughput compared to those in the BASE, DSC-UL, and DSC-DL cases.
- By combining the SPC scheme with the DCA scheme, INTD(DCA&SPC) yielded outstanding performance in terms of DL throughput, and it achieved the highest total throughput among all existing mechanisms.

**Figure 8:**
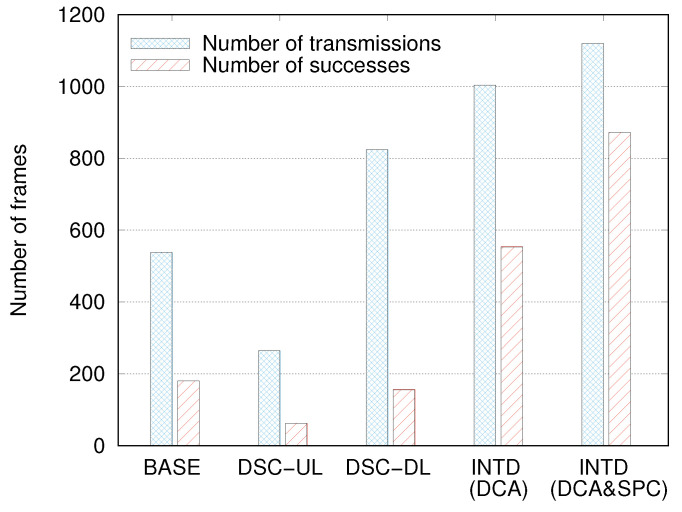
Comparison of number of frames transmitted by access point with number of frames successfully delivered to stations.

We can analyze how INTD(DCA&SPC) improved the DL throughput by observing the number of frames transmitted by the AP ($N_{DL}^{TX}$) and the number of frames successfully delivered to the STAs ($N_{DL}^{SUC}$). Figure 8 compares $N_{DL}^{TX}$ and $N_{DL}^{SUC}$ for several mechanisms. Among all of the mechanisms, $N_{DL}^{TX}$ was the least in DSC-UL, and it was approximately half that in the BASE case. In the DSC-DL case, $N_{DL}^{TX}$ increased remarkably, and it was higher than those in the DSC-UL and BASE cases by approximately 3.1 and 1.5 times, respectively. These results verify that DSC-UL is biased toward UL transmission, and this bias is greatly alleviated in DSC-DL. However, the value of $N_{DL}^{SUC}$ in DSC-DL was considerably smaller than that in the BASE case, which implies that although DSC-DL is helpful for increasing the number transmission attempts for DL frames, it hardly ensures their successful transmissions. Rather, it causes more frequent transmission failures because of serious interference.

We define $R_{DL}^{SUC}$ as the ratio of the number of successful transmissions to the total number of transmissions, that is, $R_{DL}^{SUC}=N_{DL}^{SUC}/N_{DL}^{TX}$. In the BASE, DSC-UL, and DSC-DL cases, the values of $R_{DL}^{SUC}$s were 0.33, 0.24, and 0.19, respectively. Compared to DSC-DL, INTD(DCA) significantly increased $N_{DL}^{SUC}$ and $N_{DL}^{TX}$; although $N_{DL}^{TX}$ increased by only 22%, $N_{DL}^{SUC}$ increased by more than 3.5 times. However, we observed that the $R_{DL}^{SUC}$ of INTD(DCA) was approximately 0.55. Moreover, we found that SR frame transmissions rarely failed, but most failures occurred during NSR frame transmissions. This problem was mitigated considerably in INTD(DCA&SPC). The values of $N_{DL}^{TX}$ and $N_{DL}^{SUC}$ in the case of INTD(DCA&SPC) were approximately 12% and 58% higher than those in the case of INTD(DCA), respectively, and $R_{DL}^{SUC}$ increased up to 0.78 accordingly. Based on these results, we can conclude that the combination of the SPC and DCA schemes is effective for decreasing the number of transmission failures due to interference, as well as for increasing the number of DL transmission opportunities.

### 4.4. Effects of Various Network Environments

In this section, we investigate how the performance is affected by various network environments such as (i) the number of STAs per BSS ($N_{STA}$), (ii) distance between APs ($D_{AP}$), and (iii) random network topology.

**Figure 9:**
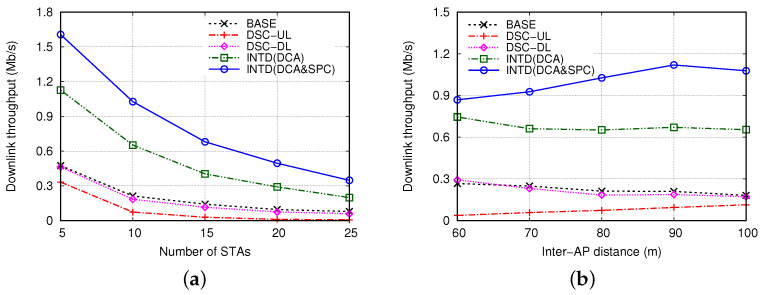
Downlink throughput with various values of (**a**) number of stations and (**b**) distance between access points.

First, Figure 9a shows the $TH_{D}$ when $N_{STA}$ was increased from 5 to 25 and $D_{AP}$ was fixed to 80 m. As $N_{STA}$ increased, $TH_{D}$ decreased in all mechanisms because of interference. For the entire range of $N_{STA}$, INTD(DCA&SPC) maintained the greatest value of $TH_{D}$; it was higher than those in the BASE and DSC-UL cases by up to 5 and 55 times, respectively, and it was higher than that in the INTD(DCA) case by 1.4–1.8 times. Similarly, the $TH_{D}$ obtained in the INTD(DCA) case was 2.4–3.1 times higher than that in the BASE case. Even though the value of $TH_{D}$ in the INTD(DCA&SPC) case decreased as $N_{STA}$ increased, it improved relative to the value in the BASE case (i.e., $TH_{D}$ in the INTD(DCA&SPC) case divided by that in the BASE case) was not affected considerably by $N_{STA}$ and was mostly higher than 4. These results indicate the excellent performance of INTD(DCA&SPC) over the existing mechanisms and its robustness to changes in the number of STAs.

Next, Figure 9b shows the effect of $D_{AP}$ on $TH_{D}$. Here, $N_{STA}$ was set to 10, and $D_{AP}$ ranged from 60 m to 100 m. Notably, the control parameters used in the proposed mechanism (i.e., $SRI_{TH}$, $CST_{SR}$ and $\Delta P_{SPC}$) were determined considering the case of $D_{AP}$ = 80 m, and they were not changed depending on the value of $D_{AP}$. The increase in $D_{AP}$ has two opposing effects: (i) the area of OBSS is narrowed and the exposed node problem is eased, such that inter-BSS interference decreases and the success probability of simultaneous DL transmissions in different BSSs increases, and on the contrary, (ii) the hidden node problem can worsen, such that the DL transmission is subject to more interference from the UL transmissions of the neighboring STAs in different BSSs. As $D_{AP}$ changes, these two effects appear in complex and different patterns in each mechanism, and the value of $TH_{D}$ in Figure 9b exhibits remarkably different trends in different mechanisms.

In the BASE case, $TH_{D}$ decreased almost linearly as $D_{AP}$ increased, implying that the second effect mentioned above was more dominant than the first one. The $TH_{D}$ values in the DSC-DL case were extremely close to those in the BASE case. As $D_{AP}$ increased, the maximum distance between the AP and the STAs in each BSS increased. Accordingly, the carrier sensing area of the AP in the DSC-DL case widened, that is, the possibility of SR decreased, which led to a decrease in $TH_{D}$ as $D_{AP}$ increased. However, the result obtained in the DSC-UL case was opposite to those obtained in the BASE and DSC-DL cases; as $D_{AP}$ increased, $TH_{D}$ increased gradually. The carrier sensing area of STA in the DSC-UL case tends to increase as $D_{AP}$ increases; thus, the asymmetry of carrier sensing between AP and STA decreases, and the AP had can have more chances for transmission, leading to an increase in $TH_{D}$. We observed that changes in $D_{AP}$ did not affect $TH_{D}$ considerably in the INTD(DCA) case; so long as $D_{AP}\geq$ 70 m, $TH_{D}$ was maintained between 0.65 Mb/s an 0.67 Mb/s, which was approximately three times higher than that in the BASE case. This result means that the performance of the proposed DCA scheme is not significantly affected by the BSS size, or it is not very sensitive to the values of the control parameters ($SRI_{TH}$ and $CST_{SR}$) determined considering the value of $D_{AP}$. This result was ascribed to the fact that, regardless of the $D_{AP}$ value, INTD(DCA) mostly succeeded in the transmission of SR frames, but it hardly succeeded in the transmission of NSR frames, which strongly supports the need for the SPC scheme. Compared to INTD(DCA), INTD(DCA&SPC) increased the $TH_{D}$ further; the $TH_{D}$ values obtained in the INTD(DCA&SPC) case were approximately 60% higher than those in the INTD(DCA) case, except when $D_{AP}$ was 60 m. The $TH_{D}$ value in the INTD(DCA&SPC) case increased as $D_{AP}$ increased up to 90 m, but it decreased when $D_{AP}$ exceeded 90 m. This result was ascribed to the two contradicting effects of $D_{AP}$ (i.e., trade-off between hidden node problem and exposed node problem), as addressed above. Compared to the BASE case, $TH_{D}$ increased by at least 3.2 times and up to 6.0 times in the INTD(DCA&SPC) case.

We performed the simulation under random network topologies. We placed 7 APs at random positions within a square area of 300 m × 300 m while maintaining $D_{AP}\geq$ 80 m. In addition, we placed the STAs randomly, such that 10 STAs were associated with each AP ($N_{STA}$ = 10). We denote $TH_{D,p\%}$ as the *p*-th percentile of $TH_{D}$ in a specific BSS and denote $G_{D,p\%}$ as the relative gain of each mechanism in terms of $TH_{D,p\%}$ compared to the BASE case, that is, the value of $TH_{D,p\%}$ in the comparative mechanism divided by that in the BASE case. Table 2 lists the $TH_{D,10\%}$, $TH_{D,50\%}$, and $TH_{D,90\%}$ values along with the $G_{D,10\%}$, $G_{D,50\%}$, and $G_{D,90\%}$ values. As summarized in Table 2, the performance of DSC-UL was comparable to that of DSC-DL, and the performance of both mechanisms were worse than that of BASE, that is, their relative gains were less than one and between 0.8 and 0.9. Even under the random topologies, INTD(DCA) and INTD(DCA&SPC) outperformed the existing mechanisms; their $G_{D,p\%}$ values were at least 2.2 and 3.5 and up to 3.3 and 6.3, respectively. Their relative gains were higher when *p* was smaller, that is, their performances were better in the worse case. Moreover, according to Table 2, the $TH_{D,10\%}$ value in the INTD(DCA&SPC) case was considerably higher than the $TH_{D,90\%}$ value in the INTD(DCA) case, which proves the advantage of the SPC scheme.

### 4.5. Fairness between SR-STAs and NSR-STAs

The increase in performance due to the proposed mechanism can be ascribed primarily to the differentiated transmission between SR-STAs and NSR-STAs, that is, transmission to SR-STAs is preferred over that to NSR-STAs. Therefore, it is necessary to investigate the fairness between SR-STAs and NSR-STAs. For this purpose, we define $N_{SR}^{TX}$ and $N_{NSR}^{TX}$ as the numbers of SR and NSR frames transmitted and $N_{SR}^{SUC}$ and $N_{NSR}^{SUC}$ as the numbers of SR and NSR frames delivered successfully, respectively.

**Figure 10:**
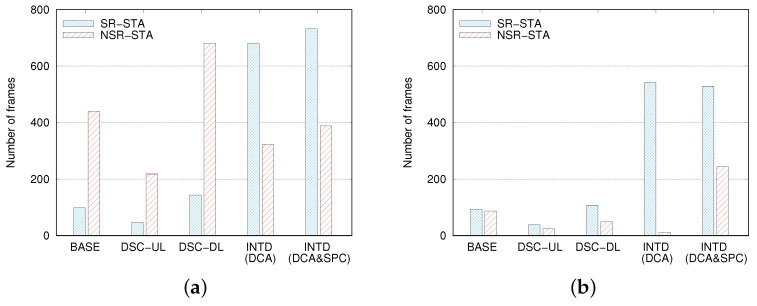
Comparison of numbers of frames (**a**) transmitted to SR-STAs and NSR-STAs and (**b**) successfully delivered to SR-STAs and NSR-STAs.

Figure 10a compares the values of $N_{SR}^{TX}$ and $N_{NSR}^{TX}$ when $N_{STA}$ = 10 and $D_{AP}$ = 80 m. Note that although there is no differentiation between SR-STAs and NSR-STAs in the existing mechanisms, we intentionally classified STAs according to the criterion described in Section 3.1 for maintaining the consistency of this comparison. In this simulation, the average number of NSR-STAs was almost twice that of SR-STAs. Therefore, $N_{NSR}^{TX}$ should be twice $N_{SR}^{TX}$ if SR-STAs and NSR-STAs are served in a completely fair manner. However, this was not the result in the BASE, DSC-UL, and DSC-DL cases; the $N_{NSR}^{TX}$ values were greater than the $N_{SR}^{TX}$ values by more than four times. We ascribe these results to the following reasons. The transmission of NSR frames is more likely to fail than that of SR frames due to interference, which results in multiple retransmissions of NSR frames. However, INTD(DCA) and INTD(DCA&SPC) yielded the opposite results, that is, $N_{SR}^{TX}$ was almost twice $N_{NSR}^{TX}$. This is because the larger CST of SR-STAs in the DCA scheme increases the number of transmissions to SR-STAs. Compared to the BASE case, INTD(DCA) and INTD(DCA&SPC) increased $N_{SR}^{TX}$ by 6.9 and 7.4 times, respectively, but they decreased $N_{NSR}^{TX}$ by 26% and 12%, respectively.

Similarly, we observed the difference between $N_{SR}^{SUC}$ and $N_{NSR}^{SUC}$. Figure 10b shows that even though $N_{NSR}^{TX}$ was considerably higher than $N_{SR}^{TX}$ in the BASE case, $N_{NSR}^{SUC}$ was rather smaller than $N_{SR}^{SUC}$. Let us define $R_{SR}^{SUC}$ = $N_{SR}^{SUC}$ / $N_{SR}^{TX}$ and $R_{NSR}^{SUC}$ = $N_{NSR}^{SUC}$ / $N_{NSR}^{TX}$. A comparison of the results in Figure 10a,b revealed that in the BASE case, $R_{NSR}^{SUC}$ was approximately 0.2, whereas $R_{SR}^{SUC}$ was almost close to 1. The unfairness between $R_{SR}^{SUC}$ and $R_{NSR}^{SUC}$ was magnified in the DSC-UL and DSC-DL cases. The values of $R_{SR}^{SUC}$ and $R_{NSR}^{SUC}$ were 0.83 and 0.11, respectively, in the DSC-UL case, and they were 0.74 and 0.07 in the DSC-DL case. From Figure 10, we can observe a serious drawback of INTD(DCA); $R_{SR}^{SUC}$ is approximately 0.8, but $R_{NSR}^{SUC}$ is less than 0.04. This means that INTD(DCA) rarely succeeds in the transmission of NSR frames, and throughput improvement is achieved by the aggressive and successful transmission of SR frames. This problem was solved to a great extent in the INTD(DCA&SPC) case, where $R_{SR}^{SUC}$ was maintained as 0.72, while $R_{NSR}^{SUC}$ was increased up to 0.63, which was greater than that in the BASE and INTD(DCA) cases by more than 3 and 16 times, respectively. Moreover, there was no significant unfairness between $R_{SR}^{SUC}$ and $R_{NSR}^{SUC}$ in the INTD(DCA&SPC) case. It is noteworthy that there exists an inevitable trade-off between improving throughput and maintaining fairness and throughput can be increased by means of SR. Thus, it is nearly impossible to increase the overall throughput while assuring fairness in terms of per-STA throughput. From the results in Figure 10, we can conclude that the SPC scheme is required for the successful transmission of NSR frames, and it contributes significantly to fairness between SR-STAs and NSR-STAs in terms of the probability (or ratio) of successful transmissions rather than that in terms of the number of transmissions.

## 5. Conclusions

We proposed the INTD mechanism to improve the DL throughput by means of SR in dense WLANs. The proposed mechanism consists of two schemes, namely DCA and SPC, and it divides STAs into SR-STAs and NSR-STAs. It differentiates the transmissions of SR and NSR frames in two aspects, channel access and transmission power. The DCA scheme preferentially serves SR frames to NSR frames by differentiating CST, while the SPC scheme selectively increases the transmission power of NSR frames. In this manner, the former scheme increases the chance of simultaneous transmission in different BSSs, and the latter scheme contributes to the successful transmission of NSR frames. The INTD mechanism can be implemented simply without any complex calculations or significant signaling overheads. The results of the simulations performed in various environments confirmed the outstanding performance of the proposed mechanism compared to that of the existing mechanisms. It increased the DL throughput by more than several times without decreasing the total throughput and maintained fairness between SR-STAs and NSR-STAs in terms of the ratio of successful transmission. We expect that the performance of the proposed INTD mechanism can be further improved by combining it with the rate adaptation mechanism, which we will investigate in a future study. Moreover, we plan to extend the proposed mechanism by considering new features of the next-generation WLAN (IEEE 802.11be) such as multi-link operation and multi-AP cooperation.

## Figures and Tables

**Table 1:** Simulation parameters.

Parameter	Value
Simulation time	10 s
Channel frequency	5.3 GHz
Channel bandwidth	20 MHz
Frame size	1472 bytes
MCS	QPSK 3/4
Transmission rate	24 Mb/s
Transmission power	25 dBm
Beacon interval	100 ms
Minimum contention window	7
Maximum contention window	1023
$SRI_{TH}$	13 dB
$CST_{NSR}$, $CST_{SR}$	−82 dBm, −67 dBm
$\Delta P_{SPC}$	10 dBm

**Table 2:** Downlink throughput and its relative gain over the BASE mechanism under random network topology.

Mechanism	Downlink Throughput (Mb/s)			Relative Gain		
	${\mathrm{TH}}_{D,10\%}$	${\mathrm{TH}}_{D,50\%}$	${\mathrm{TH}}_{D,90\%}$	$G_{D,10\%}$	$G_{D,50\%}$	$G_{D,90\%}$
BASE	0.19	0.37	0.53	N/A		
DSC-UL	0.15	0.30	0.46	0.78	0.81	0.86
DSC-DL	0.16	0.30	0.48	0.86	0.81	0.91
INTD(DCA)	0.62	1.00	1.15	3.31	2.74	2.18
INTD(DCA&SPC)	1.20	1.67	1.87	6.34	4.56	3.52

## Data Availability

Not applicable.
